# Sodium-Glucose-Cotransporter-2 Inhibitor Therapy and Intermitted Fasting in Cardiorenal Syndrome: The Role of Glucose-Mediated Oxidative Stress

**DOI:** 10.3390/jcm14030746

**Published:** 2025-01-24

**Authors:** Katrin Schröder

**Affiliations:** 1 Institute of Cardiovascular Physiology, Medical Faculty, Goethe University, 60590 Frankfurt, Germany; schroeder@vrc.uni-frankfurt.de; Tel.: +49-(0)69-6301-83660; Fax: +49-(0)69-6301-7668; 2 German Center of Cardiovascular Research (DZHK), Partner Site Rhein-Main, 60590 Frankfurt, Germany

**Keywords:** cardiorenal syndrome, SGLT2 inhibitors, intermittent fasting, glucose-mediated oxidative stress, diabetes mellitus, oxidative stress, cardiorenal protection, insulin sensitivity, metabolic health

## Abstract

Cardiorenal syndrome (CRS) is a complex clinical disorder characterized by the interplay between heart and kidney dysfunction. This condition is exacerbated by comorbidities such as diabetes mellitus, which contribute to glucose-mediated oxidative stress, further complicating the management of CRS. The management of CRS has evolved with the discovery of sodium-glucose-cotransporter-2 (SGLT2) inhibitors, which have been established as effective agents in reducing hyperglycemia and demonstrated cardiorenal protective effects. Concurrently, intermittent fasting has gained attention as an intervention without pharmacological treatment for its metabolic benefits, including improved glucose metabolism and insulin regulation and sensitivity, both with a potential reduction in oxidative stress. This review provides a summary of current findings on the roles of SGLT2 inhibitors and intermittent fasting in managing CRS, with a particular focus on glucose-mediated oxidative stress. We evaluate the mechanisms by which these interventions exert their effects, identify gaps in current research, and offer recommendations for future studies. While both SGLT2 inhibitors and intermittent fasting demonstrate potential in managing CRS, more research is needed to elucidate their long-term efficacy, safety, and potential synergistic effects.

## 1. Introduction

Cardiorenal syndrome (CRS) is a multifaceted disorder characterized by the bidirectional interaction between cardiac and renal dysfunction. The earliest mention of the term cardiorenal syndrome (CRS) came about in a 2004 National Heart, Lung, and Blood Institute Working Group conference evaluating the interaction between the heart and kidney. The term is used to commonly refer to the collective dysfunction of the heart and kidneys resulting in a cascade of feedback mechanisms causing damage to both the organs [1]. The pathophysiology of CRS involves complex neurohormonal, hemodynamic, and metabolic mechanisms, leading to a progressive decline in both cardiac and renal function [2]. Often, the causal relationship (cardiorenal vs. renocardiac) is not ascertainable, especially because risk factors like diabetes, hypertension, and atherosclerosis affect the function of both organs in parallel and the interaction of both organs with each other is complex, leading to a common clinical picture [3]. Among the contributing factors, diabetes mellitus plays a significant role in the exacerbation of CRS due to chronic hyperglycemia and increased oxidative stress [1]. The resulting glucose-mediated oxidative stress contributes to endothelial dysfunction, inflammation, and tissue damage, further complicating the management of CRS [4].

Recent advances in the treatment of CRS have highlighted the potential of novel therapeutic approaches, such as sodium-glucose-cotransporter-2 (SGLT2) inhibitors and intermittent fasting, in mitigating oxidative stress and improving clinical outcomes. SGLT2 inhibitors are a class of oral antidiabetic medications. Initially developed as glucose-lowering agents, SGLT2 inhibitors have demonstrated significant cardiorenal protective effects beyond their glycemic control capabilities [5]. By promoting urinary glucose excretion, SGLT2 inhibitors reduce hyperglycemia, lower blood pressure, and decrease oxidative stress, thereby improving cardiorenal outcomes.

Intermittent fasting, a dietary approach characterized by alternating periods of fasting and eating, has gained popularity for its potential metabolic benefits [6]. Intermittent fasting has been shown to improve insulin sensitivity, enhance mitochondrial function, and reduce oxidative stress, suggesting its potential role in managing CRS [7,8]. These effects suggest potential benefits for patients with CRS, particularly in managing glucose-mediated oxidative stress.

In general, the term “oxidative stress” is introduced to define a state between elevated levels of reactive oxygen or nitrogen species (ROS/RNS) and a decreased level of antioxidants [9]. Reactive oxygen species include the superoxide anion (·O_2_^−^), hydroxyl radical (·OH), hydrogen peroxide (H_2_O_2_), and hypochlorous acid, and reactive nitrogen species include nitric oxide (·NO), nitrogen dioxide (·NO_2_^−^) and peroxynitrite (OONO^−^). The source of production of these reactive species can be either exogenous (ionizing radiation, ultraviolet light, smoke, heavy metal ions such as Fe, Cu, Cd, Ni, and As, ozone, air pollution, etc.) or endogenous (mitochondrial dysfunction, peroxisomes, dual oxidase, lipoxygenase, cyclooxygenase) [10]. While exogenous formation of ROS often occurs in an uncontrolled manner, the intracellular mechanism for ROS formation can involve mitochondria or a specialized family of enzymes: NADPH oxidases with spatial distribution and specific signaling [11]. Importantly, ROS are not detrimental per se, but rather have dose-dependent effects [12]. Being free radicals, ROS and RNS, with their available electrons, react with other biomolecules such as lipids, proteins, and nucleic acids (DNA and RNA). The duality of free radical species in biological systems signifies that, at an optimum level, they play a crucial role in cellular processes such as defense against infectious agents, induction of a mitogenic response, and cellular structure maturation, whereas their elevated level causes a morbid effect [13,14]. Elevation in these ROS/RNS leads to oxidative stress and ultimately results in deleterious effects on cells [15].

This is a review of the existing literature on the role of SGLT2 inhibitors and intermittent fasting in the context of CRS, focusing on their impact on glucose-mediated oxidative stress. It highlights current advances, identifies gaps, and provides recommendations for future research, aiming to optimize therapeutic strategies for this complex syndrome.

## 2. Pathophysiology of Cardiorenal Syndrome

Cardiorenal syndrome is a complex disorder involving bidirectional interactions between the heart and kidneys [16]. Subtypes of CRS are classified based on their acuity and the presence of a systemic (non-cardiac, non-renal) illness. The classification of CRS into five subtypes reflects the primary organ involved and the temporal sequence of organ dysfunction [1]:

Type 1 (Acute Cardiorenal Syndrome): Acute cardiac dysfunction leads to acute kidney injury.

Type 2 (Chronic Cardiorenal Syndrome): Chronic cardiac dysfunction leads to progressive chronic kidney disease (CKD).

Type 3 (Acute Renocardiac Syndrome): Acute kidney injury leads to acute cardiac dysfunction.

Type 4 (Chronic Renocardiac Syndrome): Chronic kidney disease leads to chronic cardiac dysfunction.

Type 5 (Secondary Cardiorenal Syndrome): Systemic conditions, such as sepsis or diabetes, cause simultaneous cardiac and renal dysfunction.

## 3. Key Pathophysiological Mechanisms

Three major pathophysiological mechanisms have been suggested to promote CRS. They are: **Neurohormonal Activation:** Activation of the renin-angiotensin-aldosterone system (RAAS) and sympathetic nervous system results in vasoconstriction and sodium retention, with a simultaneous increase in blood volume and hypertension. This leads to increased cardiac workload and renal hypoperfusion, exacerbating both cardiac and renal dysfunction. Although acute kidney injury can be defined by an increase in serum creatinine by ≥0.3 mg/dL (≥26.5 micromol/L) in a 48 h period [17], there are concerns about the utility of serum creatinine as a biomarker to diagnose acute kidney injury in persons with heart failure. Rather, some of the cardiology literature refers to this as worsening renal function (WRF) [18] because it is not clear if there is evidence of kidney injury with every creatinine elevation and often the rise in creatinine is thought to be a hemodynamic effect [19]. Importantly, patients suffering from heart failure often present with low muscle mass and low protein intake. Accordingly, errors in estimated glomerular filtration rate may occur and result in false treatments and patient care. Chronic kidney disease is often associated with albuminuria, which is not necessarily the case in patients suffering from CRS. Especially in patients suffering from both, heart failure and CRS, albuminuria may not be the result of chronic kidney disease, but actually reflects cardiac dysfunction [20].

**Hemodynamic Alterations:** Reduced cardiac output and increased central venous pressure contribute to renal hypoperfusion, impairing renal function [21]. In a study involving 40 patients with acute decompensated heart failure (ADHF), 60% were found to have elevated intra-abdominal pressure (IAP), and those with IAP ≥ 8 mmHg had higher serum creatinine levels compared to those with normal IAP [22]. Conversely, renal dysfunction leads to fluid overload, high blood pressure and increased cardiac workload, worsening cardiac function [23,24]. This creates a vicious cycle where both organs deteriorate over time [25]. The role of ROS in this aspect of CRS is at least in part reflected by a study of Marenzi et al., who suggest a role of the antioxidant N-acetylcysteine in preventing contrast-induced nephropathy in patients undergoing primary angioplasty for acute myocardial infarction [26]. 

### Oxidative Stress and Inflammation

The renin-angiotensin-aldosterone-system (RAAS) not only controls blood pressure and fluid balance as mentioned above. Its activation can lead to increased production of ROS. A recent study by Su et al. suggested a role of the RAAS in salt-induced hypertension as well [27]. The resulting oxidative stress is implicated in the pathogenesis of cardiovascular diseases, including hypertension and heart failure. Studies have shown that angiotensin II, a key component of the RAAS, stimulates NADPH oxidase activity, resulting in enhanced ROS generation [28]. This oxidative stress can damage endothelial cells and promote inflammation, further exacerbating cardiovascular dysfunction [29].

Hemodynamics significantly influences the production of ROS through the dynamics of blood flow, particularly in terms of laminar and turbulent flow. Laminar flow, characterized by a smooth and orderly movement of blood, typically results in lower shear stress on the endothelial cells, leading to a balanced production of ROS [30]. In contrast, turbulent flow, which occurs at higher velocities and irregular patterns, increases shear stress and can lead to endothelial dysfunction and elevated ROS/RNS production [31]. Increased mechanical pressure and the stretching of the vessel wall can enhance the production of ROS by activating various signaling pathways in endothelial cells, which can lead to oxidative stress [32]. One mechanism involved is the activation of mechanosensitive ion channels and receptors, contributing to vascular remodeling and inflammation [33]. Besides those important constrains, chronic hyperglycemia in diabetes mellitus leads to the production of reactive oxygen species (ROS) [34] and advanced glycation end-products (AGEs) [35]. By promoting oxidative stress and inflammation, these processes contribute to endothelial dysfunction, tissue damage, and progression of CRS.

## 4. Role of Glucose-Mediated Oxidative Stress

Several studies have investigated the molecular mechanisms of glucose-mediated oxidative stress, including the activation of various signaling pathways, such as the NF-κB pathway, which leads to the production of pro-inflammatory cytokines [36]. Hyperglycemia can also lead to the activation of the polyol pathway, which results in the accumulation of sorbitol and fructose, leading to oxidative stress [37].

Glucose-mediated oxidative stress has been implicated in various diseases, including diabetes, non-alcoholic fatty liver disease (NAFLD), and cardiovascular disease. Several studies have investigated the association between glucose-mediated oxidative stress and oxidative stress markers, including 8-hydroxy-2′-deoxyguanosine (8-OHdG), malondialdehyde (MDA), and F2-isoprostanes [38]. These markers have been shown to be elevated in patients with diabetes and NAFLD, indicating increased oxidative stress.

In diabetes, glucose-mediated oxidative stress can lead to the development of complications, such as nephropathy, retinopathy, and neuropathy [39]. In NAFLD, glucose-mediated oxidative stress can contribute to the progression of the disease, leading to fibrosis and cirrhosis [40]. Given the past and future rise in the number of diabetic patients [41], there is a strong need to better understand its impact on the heart and kidneys.

## 5. Pathophysiology of Glucose-Mediated Oxidative Stress in CRS

Glucose-mediated oxidative stress is thought to play a key role in the development and progression of CRS. Hyperglycemia can lead to the production of reactive oxygen species (ROS), which can damage cellular components, including DNA, proteins, and lipids [39]. This oxidative stress can lead to inflammation, fibrosis, and apoptosis in both the heart and kidneys, contributing to the development of CRS [42].

Several clinical studies have investigated the relationship between glucose-mediated oxidative stress and CRS. A study by Cheema et al. [43] found that patients with acute heart failure had higher levels of oxidative stress markers, including 8-OHdG, compared to controls. Ronco et al. [2] found that patients with CRS had higher levels of oxidative stress and inflammation markers, including C-reactive protein (CRP) and interleukin-6 (IL6), compared to patients with heart failure alone.

Animal models have also been used to study the relationship between glucose-mediated oxidative stress and CRS. A study by Rangaswami et al. [44] found that mice with diabetes had increased oxidative stress and inflammation in the heart and kidneys, leading to the development of CRS. Another study by Palazzuoli et al. [45] connected heart failure in rats with increased oxidative stress and fibrosis in the kidneys, leading to the development of CRS.

In conclusion, glucose-mediated oxidative stress is thought to play a key role in the development and progression of CRS. Hyperglycemia can lead to oxidative stress, inflammation, and fibrosis in both the heart and kidneys, contributing to the development of CRS. Several mechanisms have been proposed to explain the role of glucose-mediated oxidative stress in CRS. These include:

1. Activation of the polyol pathway, leading to the accumulation of sorbitol and fructose, which can contribute to oxidative stress.
2. Activation of the NF-κB pathway, leading to the production of pro-inflammatory cytokines.
3. Increased production of ROS, leading to damage to cellular components.

Glucose-mediated oxidative stress plays a crucial role in the progression of CRS, highlighting the need for therapeutic strategies that effectively manage hyperglycemia and reduce oxidative stress. SGLT2 inhibitors and intermittent fasting offer potential benefits in this regard, as they target hyperglycemia and oxidative stress through distinct mechanisms.

## 6. Sodium-Glucose-Cotransporter-2 Inhibitors

The idea that mineralocorticoid receptor antagonists, such as spinolactone, could play an important therapeutic role in both heart failure and diabetic nephropathy has been discussed for quite a while. Unfortunately, male patients, in particular, suffer from unpleasant side effects such as gynecomastia, loss of libido, and impotence. With finerenone, the first non-steroidal mineralocorticoid receptor antagonist that is not intended to cause endocrine side effects, was approved in 2022. The study, FIDELITY pooled data from two phase III trials to examine the effects of finerenone on cardiovascular and kidney outcomes in patients with type 2 diabetes and chronic kidney disease. Treating patients with finerenone was associated with a significant reduction in the risk of heart attack and stroke. The researchers concluded that finerenone provides benefits in terms of kidney and cardiovascular outcomes in patients with type 2 diabetes and CKD [46].

Weight loss can effectively reduce the risk of atrial fibrillation, type 2 diabetes, and chronic kidney disease, while sodium-glucose cotransporter 2 inhibitors can reduce body weight. SGLT2 inhibitors inhibit the SGLT2 protein in the kidneys, thereby reabsorbing glucose back into the bloodstream. By blocking this protein, SGLT2 inhibitors promote the excretion of glucose through urine, thereby lowering blood glucose levels [47]. The first SGLT2 inhibitor, canagliflozin, was approved by the FDA in 2013, marking a significant milestone in diabetes treatment [48]. Subsequent inhibitors, such as empagliflozin and dapagliflozin, followed, each demonstrating unique benefits and safety profiles. Then it was discovered that hospitalization occurs less often in patients under gliflozin therapy [49]. In fact, proof of concept studies showed that SGLT2 inhibitors not only lower blood glucose levels but also provide cardiovascular and renal protective effects [50]. These findings were pivotal in establishing the broader therapeutic potential of this drug class beyond glycemic control. In a meta-analysis including 59 studies involving 108,026 patients, of whom 60,097 received gliflozins and 47,929 received a placebo, the authors found that dapagliflozin use was associated with a significant reduction in the risk of suffering from atrial fibrillation, whereas other gliflozins did not significantly prevent the disease [51]. Clinical trials have demonstrated that SGLT2 inhibitors lead not only to weight loss but also to a reduction in blood pressure, making them a valuable option for patients with type 2 diabetes who are also overweight or hypertensive [52]. Lowering blood pressure reduces the risk of heart failure and chronic kidney disease progression. A possible explanation for the effect of SGLT2 inhibitors on blood pressure is that more fluid volume is excreted due to an increased osmotic pressure established by high urine glucose levels. When the body excretes excess water, blood volume decreases and the heart does not have to pump as hard. A whole series of such effects may lead to the beneficial effects of SGLT2 inhibitors on heart failure.

Additionally, SGLT2 inhibitors change the entire metabolism [53]. Terami et al. investigated the effects of long-term treatment with the SGLT2 inhibitor, dapagliflozin, on glucose homeostasis and diabetic nephropathy in db/db mice. Treatment with dapagliflozin significantly improved glucose tolerance, reduced body weight and renal inflammation, increased triglyceride levels in the treated mice, and reduced fasting plasma glucose levels compared to vehicle-treated mice [54]. Accordingly, although reduced tubule-glomerular feedback loops have most often been considered responsible for the majority of the observed effects, they could also be attributed to a simple reduction in glucose and subsequent advanced glycosylation end-products (AGEs) [55]. AGEs are harmful compounds that form when protein or fat molecules are exposed to sugar molecules, leading to oxidative stress and inflammation. The accumulation of AGEs has been implicated in the development of various diseases, including diabetes, cardiovascular disease, and kidney disease, as they can damage tissues and disrupt normal cellular function [56]. AGEs can also stimulate the production of reactive oxygen species (ROS), creating a vicious cycle of oxidative stress and inflammation that can exacerbate disease progression. SGLT2 inhibitors reduce AGEs levels, which may contribute to their therapeutic benefits [57].

## 7. Intermitted Fasting

Intermittent fasting has been shown to have a positive impact on glucose metabolism, improving insulin sensitivity and reducing glucose levels [58]. Intermitted fasting has been found to increase the expression of genes involved in glucose metabolism, such as GLUT4, in skeletal muscle [59]. Additionally, intermitted fasting has been shown to reduce inflammation and oxidative stress, which are major contributors to insulin resistance and impaired glucose metabolism [60]. The effects of intermitted fasting on glucose metabolism are thought to be mediated by the activation of cellular pathways such as AMP-activated protein kinase (AMPK) and sirtuin 1 (SIRT1) [61]. AMPK is a key energy sensor that plays a crucial role in regulating glucose and lipid metabolism, and its activation has been shown to improve insulin sensitivity and glucose uptake in skeletal muscle [62]. SIRT1, on the other hand, is a deacetylase that regulates various cellular processes, including glucose metabolism, and its activation has been shown to improve insulin sensitivity and glucose metabolism by regulating the expression of key genes involved in glucose metabolism [63]. The activation of AMPK and SIRT1 by intermitted fasting has been shown to increase the expression of genes involved in glucose metabolism, such as GLUT4 and PPARγ, and to improve insulin signaling and glucose uptake in skeletal muscle [64].

The tumor suppressor protein p53 is activated in adipocytes in response to intermitted fasting, and plays a crucial role in regulating the immune cell landscape in adipose tissue [65]. Specifically, p53 coordinates the response to intermitted fasting by regulating the infiltration and activation of immune cells, including regulatory T cells (Tregs) and M2 macrophages, into adipose tissue. It further modulates the production of pro-inflammatory and anti-inflammatory cytokines and eventually controls the expression of genes involved in lipid metabolism, inflammation, and immune response in adipocytes. Accordingly, patients with certain mutations of p53 or p53-deficient mice are unable to properly respond to intermitted fasting. They exhibit impaired glucose tolerance, increased inflammation, and altered immune cell profiles in adipose tissue.

Very recently, arachidonic acid has been proposed to play a role in the anti-inflammatory mechanism of fasting in general. A group of 21 volunteers ate a 500 kcal meal and then fasted for 24 h before consuming a second 500 kcal meal. Restricting thier calorie intake increased blood levels of arachidonic acid and as soon as individuals ate a meal again, their levels of arachidonic acid dropped. Surprisingly, arachidonic acid in that model turns down the activity of the NLRP3 inflammasome, while it was previously linked with increased levels of inflammation [66].

Intermitted fasting has also been found to increase the production of ketone bodies, which can be used as an alternative energy source by the brain and other tissues, reducing glucose dependence [67]. Furthermore, intermitted fasting has been shown to improve beta-cell function and increase insulin secretion in response to glucose, leading to improved glucose tolerance [68]. Overall, the evidence suggests that intermitted fasting can be a useful therapeutic approach for improving glucose metabolism and reducing the risk of developing type 2 diabetes [69]. Importantly, while many studies report positive effects of intermitted fasting [70], doubts are appropriate [71]. According to preliminary observational data presented at an American Heart Association conference, eating in a 8-h window or less each day was linked with a 91% increased risk of death from cardiovascular disease compared with eating throughout a 12- to 16-h period [72,73]. This finding suggests that the heart and kidneys profit differently from intermitted fasting, a fact that has to be considered when applying intermitted fasting instead of or in combination with SGLT2 inhibitors.

## 8. Impact on Oxidative Stress

Beyond their glucose-lowering effects, SGLT2 inhibitors have been shown to modify the metabolism at multiple levels, including mitochondria and ketonbody usage [74]. Moreover, imaging mass spectrometry indicated an increased level of the tricarboxylic acid cycle intermediate, citrate, in the kidney cortex of diabetic mice. SGLT2 inhibition as well as calorie restriction almost completely eliminated citrate accumulation in the cortex. Furthermore, accumulation of oxidized glutathione in the cortex of the kidneys, prominent in the glomeruli, was also canceled by SGLT2 inhibition and calorie restriction. SGLT2 inhibition was found to suppress the production of reactive oxygen species in the kidneys, as SGLT2 inhibition prevented the diminished GSH (reduced glutathione) levels as well as elevated GSSG (oxidized glutathione) levels and GSSG-GSH ratios. In contrast, tubulointerstitial lesions, such as kidney hypertrophy, macrophage infiltration, and fibrosis, were ameliorated by calorie restriction but not by SGLT2 inhibition [75]. The SGLT2 inhibitor empagliflozin was found to reduce the expression of NOX2 and NOX4, which are two isoforms of NADPH oxidase, in the hearts of diabetic rats. Oshima et al. suggest that the inhibition of NOX2 and NOX4 by empagliflozin contributes to the reduction in ROS production and the subsequent decrease in oxidative stress [76]. Furthermore, the reduction in ROS formation increased the expression of antioxidant enzymes, such as superoxide dismutase and catalase. Additionally, the study showed that SGLT2 inhibition reduced the levels of advanced glycosylation end-products and 8-OHdG, which are markers of oxidative stress and DNA damage [77]. Overall, SGLT2 inhibition has antioxidant effects in the kidneys, which may contribute to its beneficial effects on glucose metabolism and kidney function in diabetes.

The mechanisms by which SGLT2 inhibitors reduce oxidative stress, besides increasing the expression of antioxidant enzymes, include an enhancement of the activity of the nuclear factor erythroid 2-related factor 2 (Nrf2) pathway [78,79]. SGLT2 inhibitors have been found to reduce the levels of markers of oxidative stress, such as 8-OHdG, malondialdehyde, and 4-hydroxynonenal, in both animal models and human studies [80]. The reduction in oxidative stress by SGLT2 inhibitors is associated with improved endothelial function, reduced inflammation, and enhanced mitochondrial function. The SGLT2 inhibitor dapagliflozin upregulated antioxidant enzymes such as superoxide dismutase, heme oxygenase-1, and NAD(P)H quinone dehydrogenase 1 (NQO1) in cardiac myoblast H9c2 cells, leading to improved mitochondrial dysfunction and reduced oxidative stress [81].

Intermittent fasting has been shown to reduce oxidative stress by increasing the expression of antioxidant enzymes, such as superoxide dismutase and catalase, and reducing the production of reactive oxygen species [82]. Intermitted fasting has also been found to increase the production of antioxidant molecules, such as glutathione, which plays a crucial role in protecting cells against oxidative damage [83]. A recent study on every-other-day (EOD) feeding investigated oxidative stress and inflammation in the liver of mice. EOD feeding decreased the abundance of markers of oxidative stress, such as 4-hydroxynonenal and nitrotyrosine (NT), reduced the expression of pro-inflammatory cytokines such as tumor necrosis factor-alpha and interleukin-1 beta, and increased the expression of antioxidant enzymes, such as superoxide dismutase and catalase. Consequently, EOD feeding reduced the expression of markers of apoptosis, such as caspase-3 and TUNEL-positive cells [84].

The reduction in oxidative stress by intermitted fasting has been shown to improve mitochondrial function and reduce inflammation, which are major contributors to chronic diseases such as cancer, diabetes, and neurodegenerative disorders [85]. Intermitted fasting has also been found to increase the expression of genes involved in the regulation of oxidative stress, such as Nrf2, which plays a key role in the activation of antioxidant enzymes [86]. Additionally, intermitted fasting has been shown to reduce the levels of oxidative stress markers, such as 8-OHdG and malondialdehyde (MDA), in both animal and human studies [87]. The reduction in oxidative stress by intermitted fasting has been shown to improve cardiovascular health by reducing the risk of atherosclerosis and improving endothelial function [88]. Overall, the evidence suggests that intermitted fasting can be a useful therapeutic approach for reducing oxidative stress and improving overall health, and may be particularly beneficial for individuals with chronic diseases characterized by high levels of oxidative stress.

## 9. Summary

A comparison of the effects of SGLT2 inhibitors and intermittent fasting on cardiorenal syndrome is provided in Table 1:

SGLT2 inhibitors enhance insulin sensitivity, reducing the metabolic burden on the body and decreasing oxidative stress [94]. Some of the glucose-lowering effects of SGLT2 inhibitors are partly independent of insulin, and may be related to increased glucagon-like peptide-1 (GLP-1) levels and enhanced glucose excretion in the urine [95]. By lowering blood glucose levels, SGLT2 inhibitors reduce the formation of AGEs and subsequent oxidative stress [96]. Eventually, SGLT2 inhibitors may improve mitochondrial function [97].

In summary, there is evidence that renal oxidative stress contributes to the pathogenesis of CRS, including the activation of inflammatory pathways, endothelial dysfunction, and fibrosis. Key sources of renal oxidative stress are NADPH oxidase, xanthine oxidase, and mitochondrial dysfunction. Accordingly, potential therapeutic strategies have been developed to target renal oxidative stress in CRS, including antioxidants, such as N-acetylcysteine, vitamin C, and vitamin E. For example, high-dose vitamin E supplementation reduced myocardial infarctions and cardiovascular disease endpoints in patients with end-stage renal disease [98]. Despite the fact that different doses were applied to the patients in the different studies, the data on the effects of antioxidants remain inconclusive. Accordingly, addressing renal oxidative stress via administration of SGLT2 inhibitors [64] may improve the management of CRS. The role of intermitted fasting in the treatment of CRS is less clear. However, via the reduction of blood glucose and associated oxidative stress, intermitted fasting may be beneficial as well. Nevertheless, there is recent evidence for an elevated risk of death from cardiac dysfunctions. In line with that, randomized clinical trials indicate that vitamin E and vitamin C supplementation has minimal effects on HF patients without renal dysfunction [99,100]. As they are known antioxidants, oxidative stress may play a more important role in kidney disease that in heart failure.

## Figures and Tables

**Table 1:** Comparison of the effects of SGLT2 inhibitors and intermittent fasting on cardiorenal syndrome.

Effect	SGLT2 Inhibitors	Intermitted Fasting
Glycemic control	Improves glycemic control by reducing glucose reabsorption in the kidneys [89]	Improves glycemic control by reducing insulin resistance and increasing insulin sensitivity [58]
Oxidative stress	Reduces oxidative stress by decreasing ROS production and increasing antioxidant enzymes [90]	Reduces oxidative stress by increasing antioxidant enzymes and reducing inflammation [82]
Inflammation	Reduces inflammation by decreasing pro-inflammatory cytokines [91]	Reduces inflammation by decreasing pro-inflammatory cytokines and increasing anti-inflammatory cytokines [60]
Cardiorenal outcomes	Reduces risk of cardiorenal events, such as heart failure and renal failure [92]	Improves cardiorenal outcomes by reducing blood pressure, improving lipid profiles, and reducing cardiovascular disease risk [93]
